# A comparison of the ClotPro system with rotational thromboelastometry in cardiac surgery: a prospective observational study

**DOI:** 10.1038/s41598-022-22119-x

**Published:** 2022-10-14

**Authors:** Ryogo Yoshii, Teiji Sawa, Hidetake Kawajiri, Fumimasa Amaya, Kenichi A. Tanaka, Satoru Ogawa

**Affiliations:** 1 Department of Anesthesiology, Kyoto Prefectural University of Medicine, Kyoto, Japan; 2 Department of Cardiovascular Surgery, Kyoto Prefectural University of Medicine, Kyoto, Kyoto, Japan; 3 Department of Pain Management and Palliative Care Medicine, Kyoto Prefectural University of Medicine, Kawaramachi Hirokoji, Kamigyo, Kyoto, 602-8566 Japan; 4 Department of Anesthesiology, University of Oklahoma Health Sciences Center, Oklahoma, USA

**Keywords:** Cardiology, Medical research

## Abstract

Viscoelastic coagulation tests have been increasingly used for hemostasis management in cardiac surgery. The ClotPro system is a novel viscoelastic device based on principles of rotational thromboelastometry. We aimed to compare ClotPro with ROTEM and plasma coagulation assays in cardiopulmonary bypass (CPB) patients. Blood samples were collected from 25 CPB patients at (1) baseline, (2) start of CPB, (3) end of CPB, and (4) end of surgery. The EX-test, IN-test, HI-test, FIB-test parameters on ClotPro were compared with corresponding ROTEM assay (EXTEM, INTEM, HEPTEM, and FIBTEM). Standard plasma coagulation assays and endogenous thrombin generation (TG) were simultaneously evaluated. Pearson correlation analyses showed moderate correlations between clotting times (CTs) (r = 0.63–0.67; p < 0.001, respectively), and strong correlations with maximal clot firmness (MCF) (r = 0.93–0.98; p < 0.001, respectively) between ClotPro and ROTEM. EX-test and IN-test MCF parameters were interchangeable with acceptable percentage errors (EX-test MCF: 7.3%, IN-test MCF: 8.3%), but FIB-test MCF (27.0%) and CT results were not (EX-test CT: 44.7%, IN-test CT: 31.4%). The correlations of PT/INR or peak TG with EX-test CTs were higher than with EXTEM CTs (PT/INR: r = 0.80 and 0.41, peak TG: 0.43 and 0.18, respectively). FIB-test MCF has strong correlation with plasma fibrinogen and factor XIII level (r = 0.84 and 0.66, respectively). ROC analyses showed that ClotPro was capable of emulating well-established ROTEM thresholds (area under curves: 0.83–1.00). ClotPro demonstrated strong correlations in MCF parameters of ROTEM in CPB patients. It may be reasonable to modify ROTEM-based transfusion algorithm pertaining to MCF parameters to establish cut-off values for ClotPro device.

## Introduction

In cardiac surgical patients, hemostatic functions are progressively disturbed due to loss of coagulation factors and activation of hemostatic system^1,2^. Several randomized controlled trials showed the effectiveness of viscoelastic testing for reducing the amount of blood loss and transfusion in cardiac surgery^3–5^. The recent blood management guidelines recommend the use of goal-directed transfusion algorithm using viscoelastic coagulation tests^6–10^. Most commonly utilized devices are rotational thromboelastometry (ROTEM^®^; TEM Innovations GmbH, Munich, Germany) and thromboelastography (TEG^®^; Haemonetics Corporation, Braintree, MA, USA).

The ClotPro system (enicor GmbH, Munich, Germany; Haemonetics Corporation) is a novel device that uses a modified viscoelastic coagulation test based on principal of rotational thromboelastometry^11–13^. For achieving more robust measurement and comprehensive coagulation management, some novel technologies are incorporated as twin bearing guidance-system, 6 multi-test channels, and 8 different assays lineup^13^. Several prospective randomized studies showed the ROTEM-based algorithm led to a reduction of allogeneic blood in patients undergoing cardiopulmonary bypass (CPB)^3–5^. The protocol development for ClotPro can be facilitated if ClotPro results were interchangeable with well-established ROTEM cut-off values^14^. To date, ClotPro has not been compared to other test systems in cardiac surgery. The main aim of this study was to compare various parameters of standard ClotPro testing with corresponding ROTEM testing in patients undergoing CPB surgery. In addition, we evaluated the relationships between those viscoelastic parameters and plasma coagulation assays including prothrombin time (PT), activated partial thromboplastin time (aPTT), fibrinogen, factor XIII (FXIII), and thrombin generation (TG) assay. Our hypothesis was that maximal clot firmness (MCF) between corresponding ClotPro and ROTEM tests would demonstrate a strong correlation (coefficient, r > 0.7) in CPB surgical patients.

## Methods

This study was conducted with approval of the institutional review board of Kyoto Prefectural University of Medicine (ERB-C-752), and all procedures followed the guidelines of the Helsinki Declaration. Whole blood samples were collected from 25 patients after informed written consent. Inclusion criteria were patients who were scheduled to undergo elective CPB surgery with > 20 years of age, normal PT, and aPTT before surgery. Patients on anticoagulants and those with hepatic dysfunction before surgery were excluded. In all patients, 300 IU/kg heparin was given before instituting CPB. During CPB, activated clotting times were maintained above 450 s (Hemochron Signature Elite; Instrumentation Laboratory). Following CPB, heparin was reversed with 3 mg/kg of protamine sulfate.

Whole blood samples were collected into 3.2% sodium citrated tubes (Venoject II 4.5 mL; Terumo, Tokyo, Japan) at following time points: (1) baseline after induction of anesthesia; (2) the start of CPB; (3) the end of CPB (before protamine administration), and (4) the end of surgery. Blood was either used immediately for whole blood viscoelastic measurements or centrifuged to obtain platelet poor plasma for plasma coagulation assays.

### Viscoelastic measurements

The whole blood viscoelasticity was evaluated using ClotPro and ROTEM Delta. ClotPro is a modified thromboelastometry which has 6 independent test channels. Clot formation is measured with a cup and a pin; the pin is stationary and the cuvette rotates, in contrast with ROTEM Delta^13^. The change in viscoelastic properties is continuously detected by the analyzer. All reagents are provided in a ready-to-use dried form containing a sponge in pipette tips with no requirement for reagent handling: ‘active tip’ system. Tests are initiated by electronically pipetting 340 μL of whole blood into a tip. We utilized 4 tests on ClotPro that correspond to the commonly used extrinsic, intrinsic, fibrin tests on ROTEM (i.e., EX-test, IN-test, HI-test, and FIB-test) (Table 1). Measurement is triggered either by tissue factor (EX-test) or by ellagic acid (IN-test). HI-test uses lyophilized heparinase for neutralizing heparin, and a significantly shortened HI-test clotting time (CT) over IN-test CT suggests residual heparin after protamine reversal. Contrary to single action by cytochalasin D with FIBTEM^15^, ClotPro measures fibrin-based viscoelasticity (FIB-test) with dual drugs, cytochalasin D and a glycoprotein IIb/IIIa inhibitor (tirofiban) for achieving exclusion of the platelet contribution in fibrin viscoelasticity^13^. Similar to EXTEM and FIBTEM with ROTEM, the EX-test and FIB-test with ClotPro contain polybrene to neutralize high concentrations of heparin during CPB. With ROTEM, all measurements were performed using 300 μL of whole blood with each reagent and CaCl_2_ (total: 340 μL). The following variables were collected: CT (s) which corresponds to the lag time before clotting, A5 (mm) and A10 (mm) which correspond to amplitude of clot firmness 5 and 10 min after CT, MCF (mm) as the maximal tensile strength of clot^16^. The subtraction of FIB-test (FIBTEM) from EX-test (EXTEM) was also collected as platelet component of clot formation.

**Table 1:** ClotPro and ROTEM assays.

Indications	Assay [activator and inhibitors]	
	ClotPro	ROTEM
Extrinsic coagulation assessment	EX-test [TF]	EXTEM [TF]
Reference range	CT: 38–65 s A5: 39–58 mm, A10: 47–64 mm, MCF: 53–68 mm	CT: 38–79 s A5: N/A, A10: 43–65 mm, MCF: 50–72 mm
Intrinsic coagulation assessment	IN-test [Ellagic acid]	INTEM [Ellagic acid]
Reference range	CT: 139–187 s A5: 32–53 mm, A10: 41–61 mm, MCF: 49–65 mm	CT: 100–240 s A5: N/A, A10: 44–66 mm, MCF: 50–72 mm
Heparin-insensitive assessment	HI-test [Ellagic acid + Heparinase]	HEPTEM [Ellagic acid + Heparinase]
Reference range	CT: 141–185 s	CT: 100–240 s
Fibrinogen and fibrin polymerization assessment	FIB-test [TF + Cytochalasin D + Tirofiban*]	FIBTEM [TF + Cytochalasin D]
Reference range	A5: 6–21 mm, A10: 7–23 mm, MCF: 9–27 mm	A5: N/A, A10: 7–23 mm, MCF: 9–25 mm

*TF* tissue factor, *CT* clotting time, *MCF* maximum clot firmness, *N/A* not available.

*Glycoprotein IIb/IIIa receptor inhibitor.

### Laboratory measurements

Platelet counts were measured in a hematology analyzer. Plasma PT, aPTT, fibrinogen, and FXIII level were measured on a coagulation analyzer (STACIA; LSI Medience Co., Tokyo, Japan) using manufacturer’s kits and directions. Fibrinogen levels were determined using a Clauss method (Thrombocheck Fib, Sysmex, Kobe, Japan). FXIII levels were determined using latex photometric immunoassay.

Endogenous TG assay was measured according to the previously published method^17^. All reagents for the assay were obtained from Diagnostica Stago (Parsippany, NJ, USA). Because TG assay is very sensitive to very low concentrations of heparin, only two sample points excluding heparin contamination were chosen (i.e., baseline, and end of surgery). Those plasma samples were treated with heparinase-I before TG measurement. Eighty microliter of platelet-poor plasma was added to wells of 96-well microtiter plate followed by 20 µL of 5 pmol/L tissue factor. To start the reaction 20 µL of the substrate-buffer solution was added and the TG reaction was monitored using a fluorescence reader. The lag times (min), time-to-peak (min), and peak levels of TG (nM) were collected.

### Statistical analysis

Based on the previous studies demonstrating the correlations between different viscoelastic measurements (r = 0.65–0.87)^18–20^, sample size calculations for r = 0.7 indicated the need for 23 subjects with α = 1% and 1 − β = 90%. Considering drop-outs, the sample size was determined to be 25 subjects with 100 measurements.

Data was expressed as median (IQR) according to nonnormal distributions with the Kolmogorov–Smirnov test. The statistical significances of the difference among groups were assessed by nonparametric Friedman test or Wilcoxon singed-rank test. A *p* value of 0.05 was considered significant. Pearson correlation coefficient was used to evaluate linear association between the ClotPro and the corresponding ROTEM variables. The strength of the correlation was interpreted based on common definition (0.00–0.19: very weak, 0.20–0.39: weak, 0.40–0.59: moderate, 0.60–0.79: strong, and 0.80–1.0: very strong). The agreement between measurements were analyzed using Bland–Altman plot. The bias was defined as the mean of the difference between 2 devices, and 95% limits of agreement (LOA) refer to 1.96 standard deviations (SD) of mean difference. Percentage error (1.96 SD/mean of reference method) was also calculated, and the percentage error not exceeding 30% was defined to indicate interchangeability of two parameters^21,22^. Receiver operating characteristic (ROC) curves were calculated for ClotPro variables to predict abnormal ROTEM parameters. The ROTEM values represent cut-off values typically used in goal-directed transfusion algorithms in cardiac surgery (EXTEM CT > 80–100 s; EXTEM A5 < 30 mm, A10 < 40 mm, MCF < 35–45 mm; FIBTEM A5 < 8 mm, A10 < 5–10 mm, MCF < 10 mm; INTEM CT > 240 s, HEPEM CT > 240 s, INTEM CT: HEPTEM CT ratio > 1.0–1.2)^3,5,23,24^, and optimal ClotPro cut-off values were obtained from the Youden *J* value. Cohen’s kappa coefficients were also calculated to measure agreement between ROTEM and ClotPro analyses. The strength of agreement was based on common definition (< 0.00: poor, 0.00–0.20: slight, 0.21–0.40: fair, 0.41–0.60: moderate, 0.61–0.80: substantial, and 0.81–1.0: almost perfect). All analyses were performed with Graph-Pad Prism (version 9; Graph-Pad Software Inc., San Diego, CA, USA) or JMP (version 16; SAS Institute Inc., Cary, NC, USA).

## Results

Patient demographic characteristics are shown in Table 2. Twenty-five patients had mildly to moderately complex procedures including valve replacements (n = 18), ascending aorta replacement (n = 4), total arch replacement (n = 1), and cardiac tumor resection (n = 2). The median CPB time was 147 (134–205) min.

**Table 2:** Patient demographics, transfusion, and postoperative blood loss (n = 25).

Age (years)	72 (61–75)
Sex (male/female)	16/9
Height (cm)	163 (152–164.5)
Weight (kg)	60 (55–67.5)
Surgery time (min)	312 (269–383)
CPB time (min)	147 (134–205)
**Intraoperative transfusion**	
RBC (U)	4 (0–8)
FFP (U)	4 (0–10)
Platelet concentrate (U)	20 (0–20)
**Total chest tube output in ICU (mL)**	
6 h	170 (115–300)
24 h	420 (270–680)
**Transfusion in ICU**	
RBC (U)	2 (0–2)
FFP (U)	2 (0–2)
Platelet concentrate (U)	0 (0–0)

Data are expressed as medians and quartiles (Quartile 1, Quartile 3). Volume of product per unit (U): Red blood cell (RBC), 140 mL/U; Fresh frozen plasma (FFP), 120 mL/U; (apheresis) Platelet concentrate, 250 mL/20U.

*CPB* cardiopulmonary bypass.

### Tissue factor-triggered viscoelastic measurements

**Figure 1:**
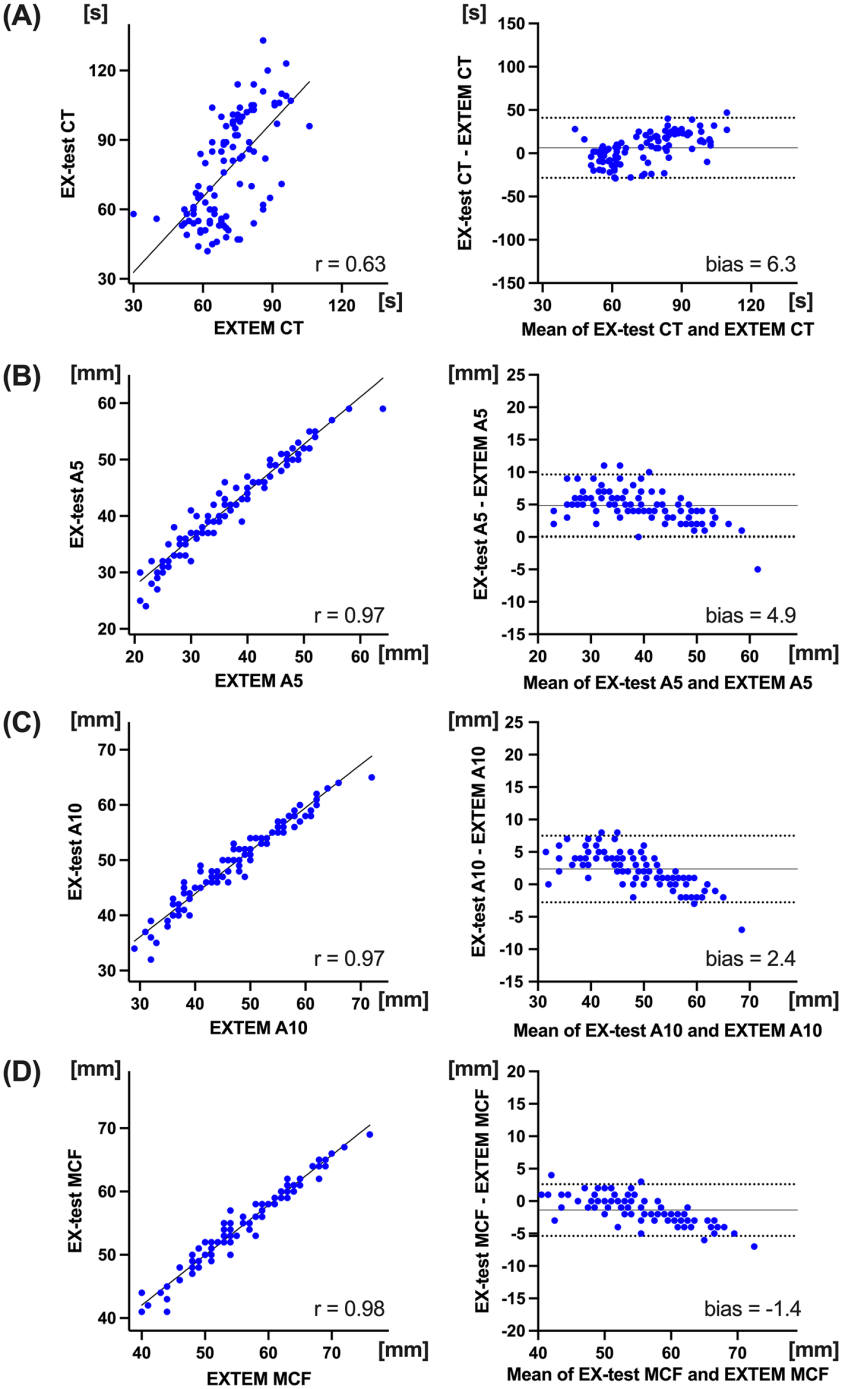
Relationship between EX-test and EXTEM. Linear regression and Bland–Altman analysis, between EX-test CT and EXTEM CT (**A**). Linear regression and Bland–Altman analysis of clot firmness, between EX-test and EXTEM; A5 (**B**), A10 (**C**), and MCF (**D**). Solid line in Bland–Altman analysis depicts bias, and dotted lines depict 95% limits of agreements. *CT* clotting time, *MCF* maximum clot firmness.

**Figure 2:**
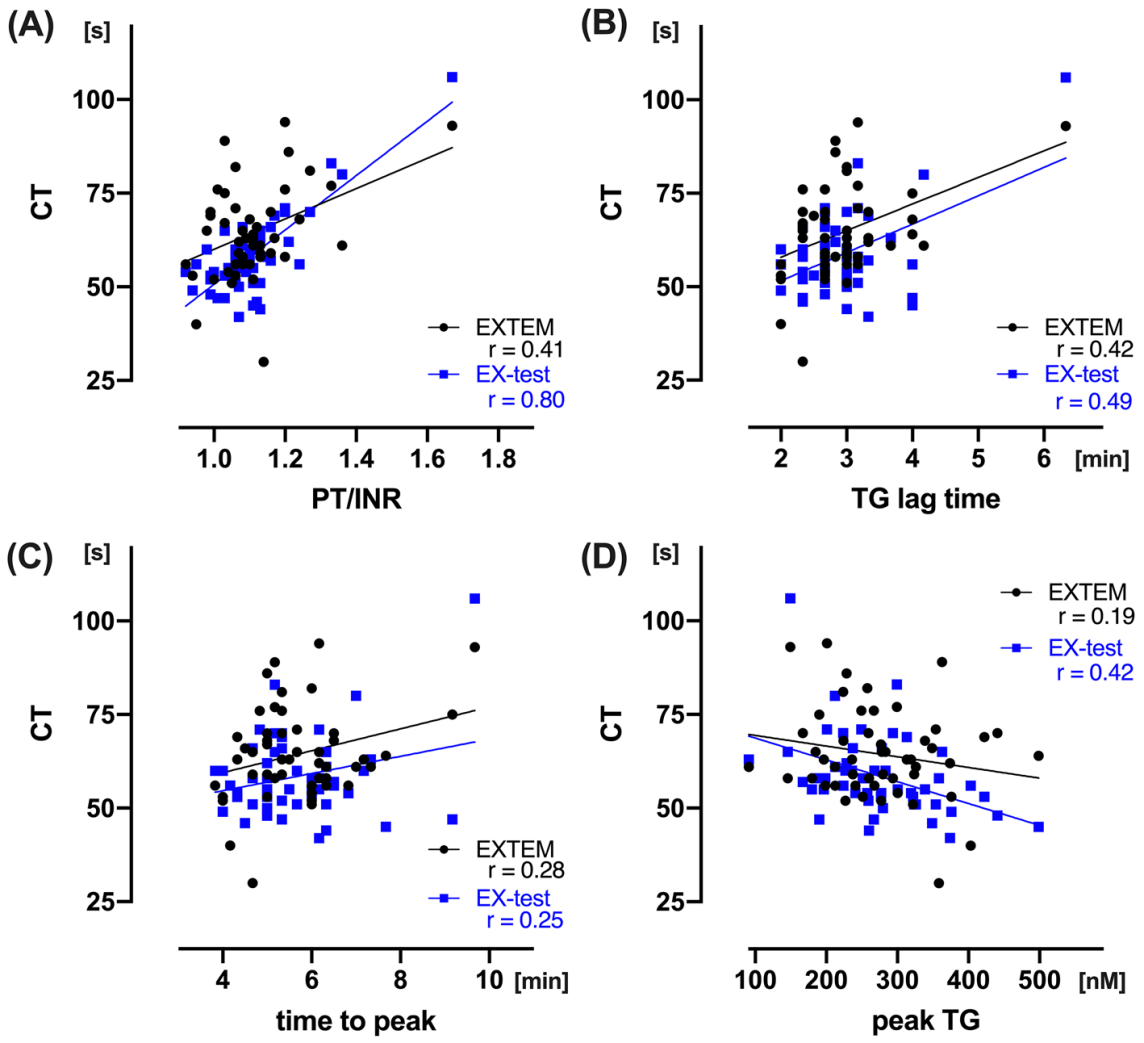
Correlation between EX-test/EXTEM CT and laboratory coagulation variables. Linear regression analysis, between EX-test/EXTEM CT and PT/INR (**A**), TG lag time (**B**), TG time to peak (**C**), and peak TG (**D**). *CT* clotting time, *PT/INR* prothrombin time/international normalized ratio, *TG* thrombin generation.

There was significant correlation between EX-test CT and EXTEM CT (r = 0.63; *p* < 0.001, Fig. 1A). Bland–Altman analysis between the CTs showed 6.3 s differences in bias (95% LOA: − 28.4 to 40.9 s), and percentage error of EX-test was 44.7%. There was no difference between two at baseline, but the CT after CPB was significantly prolonged in EX-test CT compared to EXTEM CT (104 [96–109] and 81 [74–91], respectively; *p* < 0.05). Both CTs yielded significant correlations with PT/INR, but the correlation was strong with EX-test than EXTEM (r = 0.80 and 0.41, respectively) (Fig. 2A). There were weak to moderate correlations between viscoelastic and TG parameters. Correlation between lag time and CT was strong with EX-test compared to EXTEM (r = 0.49 and 0.42, *p* < 0.005, respectively; Fig. 2B). In addition, correlation between peak TG and CT was statistically significant in EX-test (r = 0.42, *p* < 0.005), but not in EXTEM (r = 0.19, *p* = 0.18) (Fig. 2D).

The clot firmness parameter between EX-test and EXTEM showed very strong correlations (r = 0.97, 0.97 and 0.98 in A5, A10 and MCF, respectively: *p* < 0.001, respectively) (Fig. 1B–D). The MCF had a less bias (bias = − 1.4 mm) with a good agreement (95% LOA: − 5.4 to 2.6 mm), although the bias was relatively wide in A5 (bias = 4.9 mm, 95% LOA: 0.1 to 9.6 mm). The percentage error of EX-test MCF was 7.3%.

### Ellagic acid-triggered viscoelastic measurements

**Figure 3:**
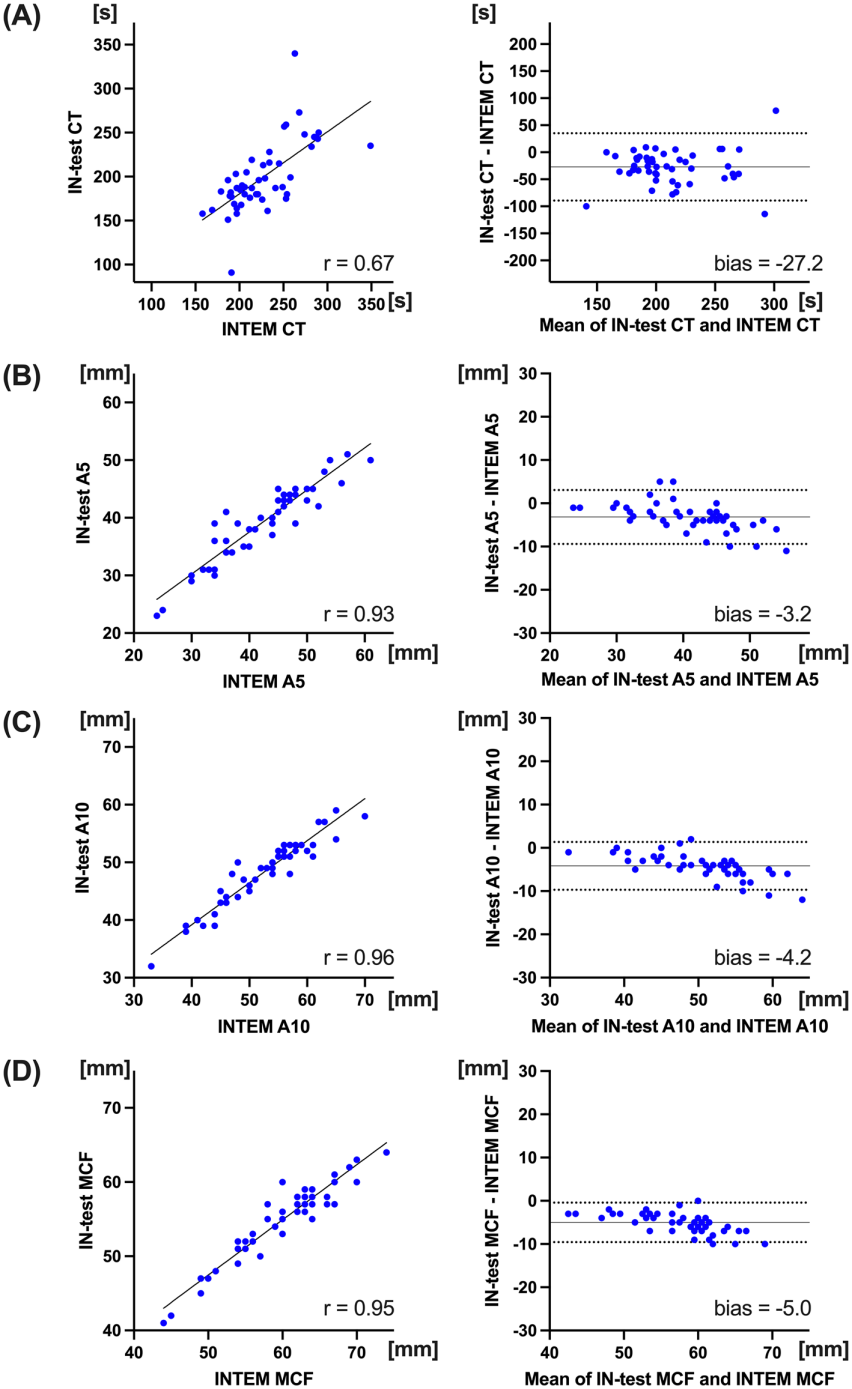
Relationship between IN-test and INTEM. Linear regression and Bland–Altman analysis, between IN-test CT and INTEM CT (**A**). Linear regression and Bland–Altman analysis of clot firmness, between IN-test and INTEM; A5 (**B**), A10 (**C**), and MCF (**D**). Solid line in Bland–Altman analysis depicts bias, and dotted lines depict 95% limits of agreements. *CT* clotting time, *MCF* maximum clot firmness.

**Figure 4:**
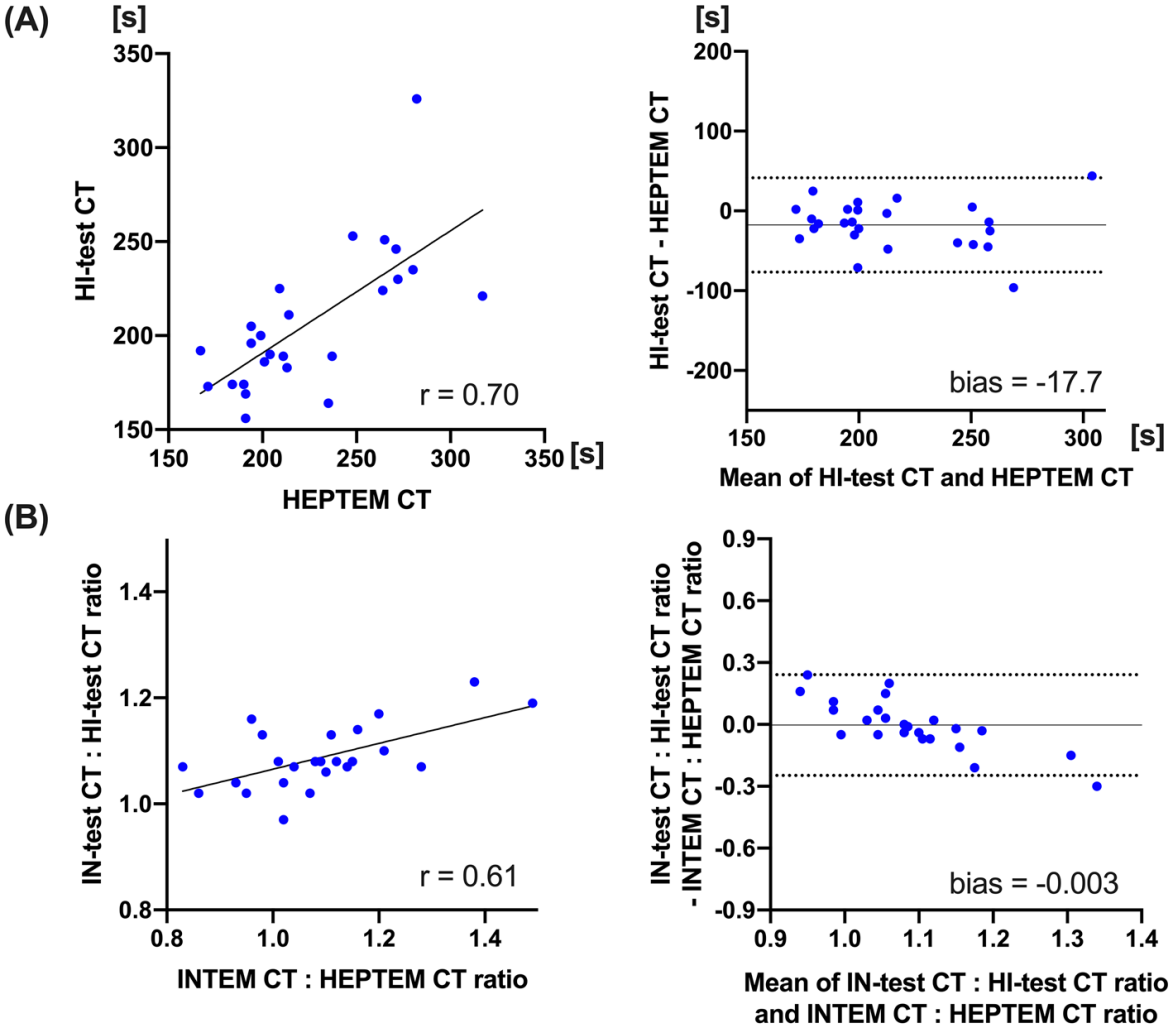
Relationship between HI-test and HEPTEM. Linear regression (**A**) and Bland–Altman analysis (**B**), between HI-test CT and HEPTEM CT. Linear regression (**C)** and Bland–Altman analysis (**D**), between IN-test CT:HI-test CT ratio and INTEM CT:HEPTEM CT ratio. Solid line in Bland–Altman analysis depicts bias, and dotted lines depict 95% limits of agreements. *CT* clotting time.

There was significant correlation between IN-test CT and INTEM CT (r = 0.67; *p* < 0.001, Fig. 3A). Bland–Altman analysis between those showed − 27.2 s differences in bias (95% LOA: − 89.4 to 35.1 s), and percentage error of IN-test CT was 31.4%. There were moderate correlations between aPTT and IN-test CT, or INTEM CT (r = 0.62 and 0.65; *p* < 0.001, respectively). Bland–Altman analysis between HI-test CT and HEPTEM CT showed − 17.7 s differences in bias with wide LOA (− 76.8 to 41.4 s), whereas the mean difference between IN-test CT:HI-test CT ratio and INTEM CT: HEPTEM CT ratio showed a bias of − 0.003 (95% LOA: − 0.25 to 0.24) (Fig. 4).

There were very strong correlations in clot firmness parameters between IN-test and INTEM (r = 0.93, 0.96 and 0.95 in A5, A10 and MCF, respectively: *p* < 0.001, respectively) (Fig. 3B–D). Bias of − 5.0 mm was found between MCFs in Bland–Altman analysis (95% LOA: − 9.6 to − 0.43 mm), and percentage error of IN-test MCF was 8.3%.

### Functional fibrin viscoelastic measurements

**Figure 5:**
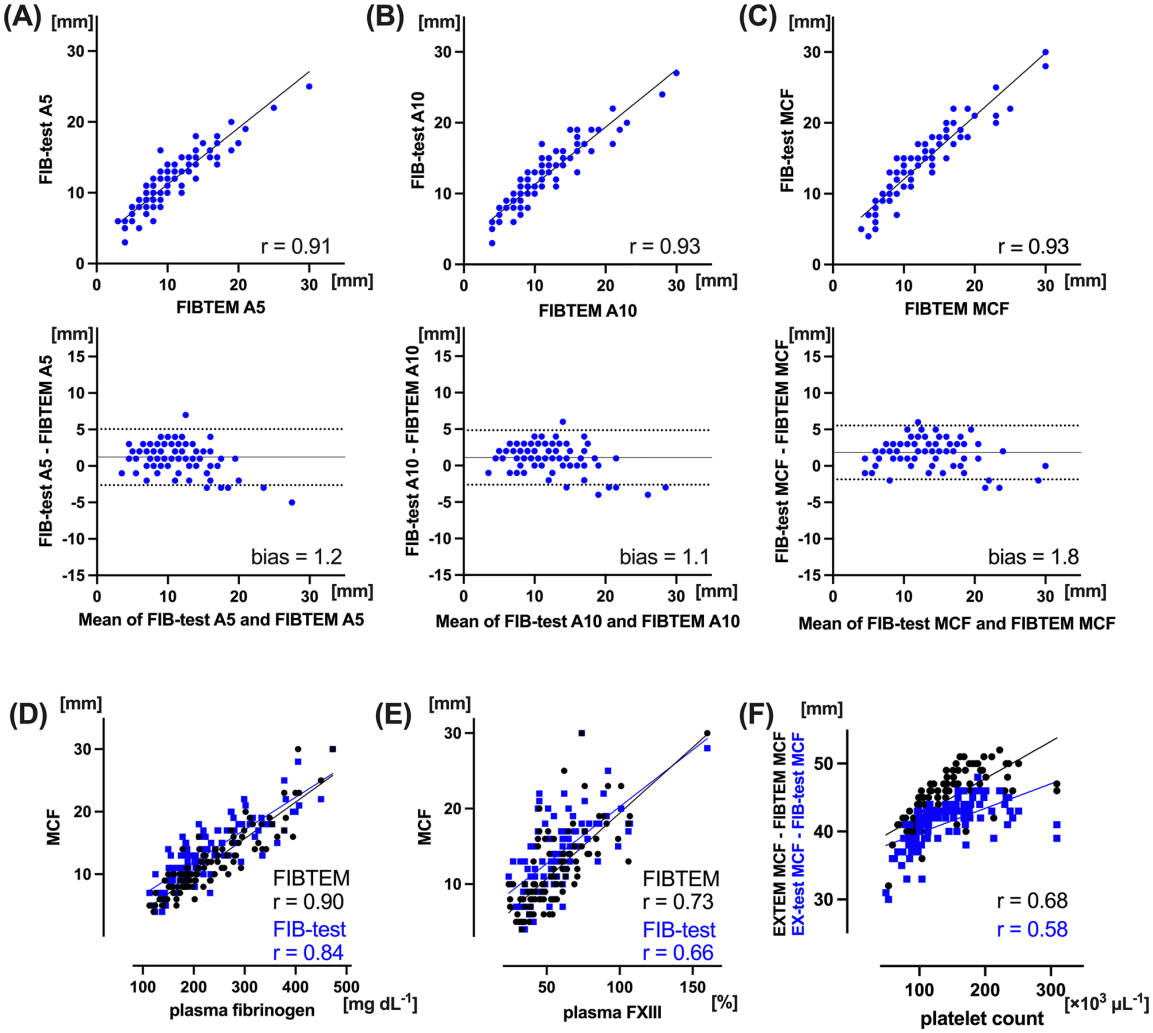
Relationship between FIB-test and FIBTEM. Linear regression and Bland–Altman analysis of clot firmness, between FIB-test and FIBTEM; A5 (**A**), A10 (**B**), and MCF (**C**). Linear regression between FIB-test/FIBTEM MCF and plasma fibrinogen (**D**), or plasma FXIII (**E**). Linear regression between platelet count and difference between EX-test MCF and FIB-test MCF, or EXTEM MCF and FIBTEM MCF (**F**). Solid line in Bland–Altman analysis depicts bias, and dotted lines depict 95% limits of agreements. *MCF* maximum clot firmness, *FXIII* factor XIII.

There were very strong correlations in clot firmness parameter between FIB-test and FIBTEM (r = 0.91, 0.93 and 0.93 in A5, A10 and MCF, respectively: *p* < 0.001, respectively) (Fig. 5A–C). Bland–Altman analysis between two MCFs showed 1.8 mm of bias with a narrow LOA (− 1.9 to 5.5 mm) (Fig. 5C), and percentage error of FIB-test MCF was 27.0%. There were very strong correlations between plasma fibrinogen levels and FIB-test MCF, or FIBTEM MCF (r = 0.84 and 0.90; *p* < 0.001, respectively; Fig. 5D). Plasma factor XIII also yield strong correlation between FIB-test MCF, or FIBTEM MCF (r = 0.66 and 0.73; *p* < 0.001, respectively; Fig. 5E). Significant correlations were found between platelet count and difference between EX-test and FIB-test, or EXTEM and FIBTEM (*r* = 0.58 and 0.68; *p* < 0.001; Fig. 5F).

### ROC analysis

The results of the ROC analyses were shown in Table 3. The cut-off values of ClotPro testing predicted specific values of corresponding ROTEM thresholds with high area under curves (0.86–1.0) and negative predictive values (0.92–1.0). Based on the kappa coefficient analyses, there was only slight agreement between EXTEM CT and EX-test CT (Κ = 0.34; p < 0.001) and moderate agreement between INTEM CT and IN-test CT (Κ = 0.62; *p* < 0.001). Furthermore, there was only slight agreement between INTEM CT:HEPTEM CT ratio and IN-test CT:HI-test CT ratio (Κ = 0.39; *p* = 0.030). Strong agreements in clot firmness parameters between EXTEM and EX-test were found (K > 0.8; *p* < 0.0001, respectively). Agreement between FIBTEM and FIB-test clot firmness parameters were only substantial (K < 0.8; *p* < 0.0001, respectively).

**Table 3:** ROC analysis of ClotPro parameters for various ROTEM thresholds.

Model	ClotPro cutoff value	AUC (95% CI)	Sensitivity (95% CI)/Specificity (95% CI)	PPV/NPV	ROTEM	ClotPro		Cohen’s K (*p* value)
EXTEM CT > 90 s versus EX-test CT	> 95 s	0.87 (0.77–0.97)	0.90 (0.71–1.00)/0.77 (0.68–0.85)	0.30/0.99		≦ 95 s	> 95 s	0.34 (*p* < 0.0001)
					≦ 90 s	68	22	
					> 90 s	1	9	
EXTEM A5 < 30 mm versus EX-test A5	< 36 mm	0.99 (0.97–1.00)	0.96 (0.88–1.00)/0.97 (0.94–1.00)	0.92/0.99		≧ 36 mm	< 36 mm	0.86 (*p* < 0.0001)
					≧ 30 mm	75	1	
					< 30 mm	4	20	
EXTEM A10 < 40 mm versus EX-test A10	< 44 mm	1.00 (0.99–1.00)	0.92 (0.82–1.00)/1.00 (1.00–1.00)	1.00/0.97		≧ 44 mm	< 44 mm	0.89 (*p* < 0.0001)
					≧ 40 mm	74	0	
					< 40 mm	4	22	
EXTEM MCF < 45 mm versus EX-test MCF	< 45 mm	1.00 (1.00–1.00)	1.00 (1.00–1.00)/1.00 (1.00–1.00)	1.00/1.00		≧ 45 mm	< 45 mm	0.85 (*p* < 0.0001)
					≧ 45 mm	92	0	
					< 45 mm	2	6	
INTEM CT > 240 s versus IN-test CT	> 228 s	0.86 (0.74–0.98)	0.63 (0.39–0.86)/1.00 (1.00–1.00)	1.00/0.84		≦ 228 s	> 228 s	0.62 (*p* < 0.0001)
					≦ 240 s	32	0	
					> 240 s	8	10	
HEPTEM CT > 240 s versus HI-test CT	> 211 s	0.99 (0.95–1.02)	1.00 (1.00–1.00)/0.94 (0.83–1.00)	0.89/1.00		≦ 211 s	> 221 s	0.91 (*p* < 0.0001)
					≦ 240 s	16	1	
					> 240 s	0	8	
INTEM CT:HEPTEM CT ratio > 1.10 versus IN-test CT:HI-test CT ratio	> 1.06	0.83 (0.67–0.99)	1.00 (1.00–1.00)/0.50 (0.24–0.76)	0.59/1.00		≦ 1.06	> 1.06	0.39 (*p* = 0.030)
					≦ 1.10	7	7	
					> 1.10	1	10	
FIBTEM A5 < 8 mm versus FIB-test A5	< 9 mm	0.94 (0.90–0.98)	0.86 (0.75–0.97)/0.88 (0.79–0.96)	0.79/0.92		≧ 9 mm	< 9 mm	0.66 (*p* < 0.0001)
					≧ 8 mm	61	3	
					< 8 mm	12	24	
FIBTEM A10 < 8 mm versus FIB-test A10	< 9 mm	0.98 (0.95–1.00)	0.96 (0.88–1.00)/0.93 (0.88–0.99)	0.82/0.99		≧ 9 mm	< 9 mm	0.71 (*p* < 0.0001)
					≧ 8 mm	73	3	
					< 8 mm	7	17	
FIBTEM MCF < 8 mm versus FIB-test MCF	< 10 mm	0.98 (0.96–1.00)	1.00 (1.00–1.00)/0.89 (0.83–0.96)	0.64/1.00		≧ 10 mm	< 10 mm	0.76 (*p* < 0.0001)
					≧ 8 mm	79	5	
					< 8 mm	2	14	

*ROC* receiver operating characteristic, *AUC* area under curve, *95% CI* 95% confidence interval, *PPV* positive predictive value, *NPV* negative predictive value, *CT* clotting time, *MCF* maximum clot firmness.

## Discussion

In this comparative study of viscoelastic measurements in cardiac surgery, we demonstrated that ClotPro parameters have strong correlations with ROTEM parameters. Our data showed that extrinsic and intrinsic MCF parameters were interchangeable with low percentage errors, but fibrin-specific MCF and CT results were not. ClotPro parameters had weak to strong correlations with various plasma coagulation assays in central laboratories. The correlation of tissue-factor triggered PT or TG assay with ClotPro was better than with ROTEM. The ROC analyses showed that ClotPro was capable of emulating well-established ROTEM thresholds in cardiac surgery. However, EXTEM CT, INTEM CT, INTEM CT:HEPTEM CT ratio, and FIBTEM clot firmness parameters showed only limited agreement with Cohen’s kappas below 0.8. Accordingly, the extrapolation of ClotPro testing results on established transfusion algorithm using ROTEM should be considered with caution.

The ClotPro is increasingly used as new viscoelastic device^11–13,25–29^, and is currently available in Europe and Japan. There was one clinical study for CPB patients using ClotPro, but the aim of study was evaluation of TPA-test alone^27^. Our study was first head-to-head ex vivo comparison of standard ClotPro tests with a well-established ROTEM assays in CPB surgery. Implementing ROTEM parameters into transfusion algorithms has been repeatedly shown to reduce blood transfusion amounts in CPB surgery^3–5^. In the randomized clinical trials with ROTEM, transfusion algorithm mainly incorporated EXTEM CT, EXTEM and FIBTEM clot firmness. Device-specific thresholds of ClotPro need to be established to implementing it in cardiac surgery. Our data suggested high agreement between EXTEM and EX-test clot firmness parameters with low percentage errors (K between 0.85 and 0.89). However, other parameters showed only slight to substantial agreement (K between 0.34 and 0.76). The biases between two devices were minimal in clot firmness parameters for extrinsic or intrinsic assay. However, the inferior agreement with a percentage error of 27% and apparent bias (bias = 1.8 mm, Fig. 5C) between FIBTEM and FIB-test MCF may affect fibrinogen replacement therapies. Our study also confirmed that the efficacy with early clot firmness of ClotPro (A5 or A10) for predicting ROTEM thresholds are comparable to with MCF.

Regarding CT, substantial variabilities were found in extrinsic and intrinsic tests between ClotPro and ROTEM devices (Figs. 1, 3). And, the variability was prominent in EX-test CT with high percentage error of 44.7%. Gillissen et al. compared various parameters between ROTEM delta and ROTEM sigma, semi-automated successor, in postpartum hemorrhage patients^30^. In their study, there was strong correlation in EXTEM MCF between two devices (r = 0.84), but no significant correlation in EXTEM CT (r = 0.18). It is speculated that multiple factors including reagent compositions, and different measurement principles affect clotting time results^13,31^. In particular, use of different tissue factor sources may lead to variation in tissue factor-triggered clotting time results, in contrast with standardized prothrombin time testing in central laboratories. Although the addition of reagent causes some extent of dilution with blood sample in ROTEM measurements, extra dilution is not accompanied with dry-reagent technology of ClotPro measurements. Indeed, the reference ranges of CTs in ROTEM assays are high compared to those in corresponding ClotPro assays (Table 1). Extra dilution in ROTEM measurement may attribute to CT results.

In addition, our data demonstrated that both viscoelastic measurements have significant correlations with standard plasma coagulation tests. The correlations between tissue-factor triggered PT and TG parameters were strong with EX-test CT compared to EXTEM CT (Fig. 2). Interestingly, the prolongation of CT after CPB was more extensive in EX-test. Those result may indicate that EX-test CT is more susceptible to decrease of prothrombin after the hemodilution^32^. Further clinical studies are needed to assess if EX-test CT is more sensitive than EXTEM CT in determining the need for transfusion in CPB surgery. Our data showed that both IN-test and HI-test CT results were significantly lower than INTEM and HEPTEM CT, respectively. Accordingly, there was no bias between IN-test:HI-test ratio and INTEM:HEPTEM ratio. It is feasible that IN-test: HI-test ratio is a clinical surrogate of ROTEM after the protamine reversal.

For aim of excluding platelet contribution in fibrin viscoelasticity, ClotPro uses tirofiban as platelet inhibitor in addition to cytochalasin D. Solomon et al. compared the effect of tirofiban combined with cytochalasin D (FIBTEM PLUS) on ROTEM-clot firmness compared to cytochalasin D alone^33^. In their study, FIBTEM MCF in the combination was significantly lower than in cytochalasin D alone at baseline sample^33^. However, our results did not show an advantage of using tirofiban in fibrin-specific viscoelastic testing. FIB-test MCF had strong correlations with plasma fibrinogen concentration and factor XIII activity (r = 0.84 and 0.66, respectively), but correlations were superior for FIBTEM (r = 0.90 and 0.73, respectively) (Fig. 5D,E). The correlation of platelet component of clot formation (using difference between extrinsic-triggered and fibrin-specific testing) with platelet count was also superior with ROTEM, compared to ClotPro (*r* = 0.68 and 0.58, respectively; Fig. 5F). Moreover, FIB-test was associated with higher clot firmness results compared to FIBTEM which may suggest even less platelet inhibition in FIB-test (Fig. 5A–C). The further investigation is required to clarify the difference between two fibrin-specific viscoelastic measurements.

Measurement process of current generation of ROTEM (ROTEM sigma) is fully-automated, and reagents consist of a consumable ready-to-use cartridge. Although pipetting is required on ClotPro device, the “active tip” system with no requirement for reagent handling may facilitate an uncomplicated procedure. ClotPro allows 6 simultaneous testing while other viscoelastic devices have normally 4 channels. Extensive test availability may cause clinical advantage from comprehensive coagulation assessment for severe coagulation depletions, preoperative anticoagulant usage, and protamine-heparin dosing in cardiac surgery.

There is a major limitation in our study. Although main purpose of this study was to evaluated the relationships between ClotPro and ROTEM, the sample numbers may be underpowered to define optimal cut-off values of ClotPro. The study was not designed to detect clinical outcomes as bleeding or blood transfusion amounts. Because the decision to transfuse hemostatic products was not based on ROTEM or ClotPro results, we are unable to comment the reduction effect of ClotPro on blood transfusion. The future interventional studies to evaluate the association between ClotPro and relevant clinical outcomes are required.

Our study has another limitation. We did not perform TPA-test for evaluating the effect of antifibrinolytics, although common dose of tranexamic acid was used for all patients. It is plausible that more comprehensive hemostatic approach could be implemented using this specific test, in parallel with standard ClotPro testing we used. Additional study is currently in progress to evaluate the efficacy of TPA-test in cardiac surgery.

In conclusion, our data indicated that ClotPro clot firmness parameters were strongly correlated with ROTEM and plasma laboratory measurements in cardiac surgical patients. The interchangeabilities of MCFs between EX-test and EXTEM assays were high, but substantial variabilities were found in CTs and FIBTEM clot firmness parameters. Additional clinical studies with large patient populations are warranted to define optimal cut-off values of ClotPro in cardiac surgery.

## Data Availability

The datasets used and/or analysed during the current study available from the corresponding author on reasonable request.

## References

[1] Bolliger D, Gorlinger K, Tanaka KA (2010). Pathophysiology and treatment of coagulopathy in massive hemorrhage and hemodilution. Anesthesiology.

[2] Ogawa S (2013). Haemodilution-induced changes in coagulation and effects of haemostatic components under flow conditions. Br. J. Anaesth..

[3] Weber CF (2012). Point-of-care testing: A prospective, randomized clinical trial of efficacy in coagulopathic cardiac surgery patients. Anesthesiology.

[4] Nakayama Y (2015). Thromboelastometry-guided intraoperative haemostatic management reduces bleeding and red cell transfusion after paediatric cardiac surgery. Br. J. Anaesth..

[5] Karkouti K (2016). Point-of-care hemostatic testing in cardiac surgery: A stepped-wedge clustered randomized controlled trial. Circulation.

[6] Kozek-Langenecker SA (2017). Management of severe perioperative bleeding: Guidelines from the European Society of Anaesthesiology: First update 2016. Eur. J. Anaesthesiol..

[7] Thomas W (2018). The utility of viscoelastic methods in the prevention and treatment of bleeding and hospital-associated venous thromboembolism in perioperative care: Guidance from the SSC of the ISTH. J. Thromb. Haemost..

[8] Boer C (2018). 2017 EACTS/EACTA guidelines on patient blood management for adult cardiac surgery. J. Cardiothorac. Vasc. Anesth..

[9] Raphael J (2019). Society of Cardiovascular Anesthesiologists Clinical Practice Improvement Advisory for management of perioperative bleeding and hemostasis in cardiac surgery patients. Anesth. Analg..

[10] Tibi P (2021). STS/SCA/AmSECT/SABM update to the clinical practice guidelines on patient blood management. J. Cardiothorac. Vasc. Anesth..

[11] Fong AYY (2020). Effect of dabigatran on clotting time in the clotpro ecarin clotting assay: A prospective, single-arm, open-label study. Clin. Appl. Thromb. Hemost..

[12] Groene P (2021). Functional testing of tranexamic acid effects in patients undergoing elective orthopaedic surgery. J. Thromb. Thrombolysis.

[13] Bareille M (2021). Viscoelastometric testing to assess hemostasis of COVID-19: A systematic review. J. Clin. Med..

[14] Bolliger D, Tanaka KA (2013). Roles of thrombelastography and thromboelastometry for patient blood management in cardiac surgery. Transfus Med. Rev..

[15] Ogawa S (2012). The impact of hematocrit on fibrin clot formation assessed by rotational thromboelastometry. Anesth. Analg..

[16] Ogawa S (2012). A comparative evaluation of rotation thromboelastometry and standard coagulation tests in hemodilution-induced coagulation changes after cardiac surgery. Transfusion.

[17] Takeshita S (2019). Prohemostatic activity of factor X in combination with activated factor VII in dilutional coagulopathy. Anesth. Analg..

[18] Huffmyer JL, Fernandez LG, Haghighian C, Terkawi AS, Groves DS (2016). Comparison of SEER sonorheometry with rotational thromboelastometry and laboratory parameters in cardiac surgery. Anesth. Analg..

[19] Baryshnikova E, Di Dedda U, Ranucci M (2019). A comparative study of SEER sonorheometry versus standard coagulation tests, rotational thromboelastometry, and multiple electrode aggregometry in cardiac surgery. J. Cardiothorac. Vasc. Anesth..

[20] DeAnda A (2021). Comparison of the quantra QPlus system with thromboelastography in cardiac surgery. J. Cardiothorac. Vasc. Anesth..

[21] Critchley LA, Critchley JA (1999). A meta-analysis of studies using bias and precision statistics to compare cardiac output measurement techniques. J. Clin. Monit. Comput..

[22] Paarmann H (2011). Lack of agreement between pulmonary arterial thermodilution cardiac output and the pressure recording analytical method in postoperative cardiac surgery patients. Br. J. Anaesth..

[23] Tanaka KA, Bolliger D, Vadlamudi R, Nimmo A (2012). Rotational thromboelastometry (ROTEM)-based coagulation management in cardiac surgery and major trauma. J. Cardiothorac. Vasc. Anesth..

[24] Gorlinger K (2019). The role of evidence-based algorithms for rotational thromboelastometry-guided bleeding management. Korean J. Anesthesiol..

[25] Bachler M (2021). Impaired fibrinolysis in critically ill COVID-19 patients. Br. J. Anaesth..

[26] Heinz C (2021). Greater fibrinolysis resistance but no greater platelet aggregation in critically ill COVID-19 patients. Anesthesiology.

[27] Kammerer T (2021). Functional testing for tranexamic acid duration of action using modified viscoelastometry. Transfus Med. Hemother..

[28] Groene P (2021). Viscoelastometry for detecting oral anticoagulants. Thromb. J..

[29] Oberladstatter D (2021). A prospective observational study of the rapid detection of clinically-relevant plasma direct oral anticoagulant levels following acute traumatic injury. Anaesthesia.

[30] Gillissen A (2019). Comparison of thromboelastometry by ROTEM((R)) delta and ROTEM((R)) sigma in women with postpartum haemorrhage. Scand. J. Clin. Lab. Investig..

[31] Judd M (2020). Clotting time results are not interchangeable between EXTEM and FIBTEM on rotational thromboelastometry. J. Cardiothorac. Vasc. Anesth..

[32] Okabayashi S (2018). A comparative study of point-of-care prothrombin time in cardiopulmonary bypass surgery. J. Cardiothorac. Vasc. Anesth..

[33] Solomon C (2013). FIBTEM PLUS provides an improved thromboelastometry test for measurement of fibrin-based clot quality in cardiac surgery patients. Anesth. Analg..

